# Outdoor Artificial Light at Night and Insomnia-Related Social Media Posts

**DOI:** 10.1001/jamanetworkopen.2024.46156

**Published:** 2024-11-20

**Authors:** Jiahao Duan, Qian Li, Zhouxin Yin, Shihan Zhen, Wenzhe Cao, Shiwei Yan, Yanhui Zhang, Qingyao Wu, Wei Zhang, Fengchao Liang

**Affiliations:** 1 School of Public Health and Emergency Management, Southern University of Science and Technology, Shenzhen, China; 2 School of Medicine, Southern University of Science and Technology, Shenzhen, China; 3 School of Safety Science and Emergency Management, Wuhan University of Technology, Wuhan, China

## Abstract

**Importance:**

Understanding the outcomes of artificial light at night (ALAN) on insomnia is crucial for public health, particularly in rapidly urbanizing regions. However, evidence of the association between ALAN exposure and insomnia is limited, despite the large number of people exposed to ALAN.

**Objective:**

To explore the association between outdoor ALAN exposure and insomnia among the Chinese population.

**Design, Setting, and Participants:**

This cross-sectional study used social media data from Weibo (Sina), a social media platform, and satellite-derived nighttime light images. The study period spans from May 2022 to April 2023. The study encompasses 336 cities across China’s mainland, providing a comprehensive national perspective. Data include insomnia-related posts from the platform users, representing a large and diverse population sample exposed to varying levels of ALAN.

**Exposure:**

Outdoor ALAN exposure (in nanowatts per centimeters squared per steradian [nW/cm^2^/sr]) was measured using satellite-derived nighttime light images at a spatial resolution of 500 m.

**Main Outcomes and Measures:**

The incidence of insomnia among residents at the city level was measured by the number of insomnia-related posts on social media. Multiple linear regression models were used to estimate the association between ALAN exposure and population insomnia, adjusting for population characteristics and meteorological factors at the city level.

**Results:**

The study included data from 1 147 583 insomnia-related posts. Daily mean ALAN exposure across the 336 cities ranged from 3.1 to 221.0 nW/cm^2^/sr. For each 5 nW/cm^2^/sr increase in ALAN exposure, the incidence of insomnia increased by 0.377% (95% CI, 0.372%-0.382%). The association was greater in less populated cities and under extreme temperature and poor air quality conditions. The observed exposure-response functions between ALAN exposure and insomnia demonstrated an upward trend, with steeper slopes observed at low exposures and leveling off at higher exposures.

**Conclusions and Relevance:**

This study provides evidence of the association between increased ALAN exposure and higher incidence of insomnia. These findings expand the current knowledge on adverse health outcomes of ALAN exposure and emphasize the potential health benefits of well-planned artificial nighttime lighting in China and other developing countries in the early stages of city planning.

## Key Points

**Question:**

Is exposure to artificial light at night (ALAN) associated with an increased risk of insomnia among the Chinese population?

**Findings:**

In this cross-sectional study, by using over 1.1 million insomnia-related social media posts and satellite-derived nighttime light images, a significant association was found between higher ALAN exposure and increased incidence of insomnia rates across 336 cities in China’s mainland.

**Meaning:**

These findings highlight the adverse health outcomes of ALAN exposure and the potential health benefits of well-planned nighttime lighting in early city planning for developing countries.

## Teaser

This cross-sectional study uses social medial posts and satellite-derived nighttime light images to explore the association between exposure to outdoor artificial light at night and insomnia among the Chinese population.

## Introduction

Light pollution, an emerging form of environmental pollutant, refers to excessive or intrusive artificial light resulting from poor lighting design.^1^ Earth’s artificially lit outdoor area exhibits a global escalation at an annual rate of approximately 2.2%,^2^ affecting the living environment of more than 80% of the global population,^3^ with a principal source being artificial light at night (ALAN).^4^ ALAN exposure is typically ascribed to regional urbanization,^5^ and its severity is increasingly pronounced. China is the country experiencing the most rapid proliferation of nighttime light, characterized by an annual growth rate of over 6%.^6^

Sleep is a vital mechanism for energy restoration and enhancing internal resistance.^7^ Compared with natural light, ALAN emits more short-wavelength blue light, which inhibits the secretion of melatonin within the human body,^8,9^ leading to a decline in sleep quality and circadian rhythm disorder.^10,11^ Elevated exposure to ALAN has been associated with a series of diseases, including insomnia, depressive disorders, and metabolic irregularities,^6,12,13,14^ which may further increase the risks of morbidity and mortality.^15,16^ Therefore, it is necessary to conduct comprehensive research on the association between ALAN and insomnia.

Existing research primarily used telephone survey and questionnaires^4,17,18,19^ to collect insomnia information, assessing the association between ALAN exposure and insomnia through cross-sectional or cohort study.^6,8,20,21^ Although these approaches used high-quality data, they may be susceptible to recall bias, time lag, and limitations in coverage when quantifying the impact of ALAN exposure on insomnia. The use of social media posts, those nearly real-time and spontaneous expressions about health conditions, could reduce possible recall bias and reporting bias, and the diverse distribution of users in different characters could represent a general population. Those active posts could be a possible way to analyze health impact on mild health symptoms such as insomnia. Social media platforms such as Twitter and Facebook, which have been widely adopted in Europe and the US, offer an alternative approach of data collection in health risk analyses.^22,23,24^ In China, Weibo (Sina) and Baidu Index are commonly used sources of social media data.^25,26,27^ Some research has used data from these platforms to construct daily city-level happiness indicators, shedding light on the association between fine particulate matter exposure and population happiness.^25,27^ This innovative approach has the potential to mitigate the limitations of traditional methods, enabling more comprehensive investigations into the association between ALAN exposure and insomnia. Incorporating satellite-based ALAN observations with daily city-level insomnia data sourced from social media, we conducted a nationwide study through machine learning and various statistical methods to assess the association between ALAN exposure and insomnia among the Chinese population.

## Methods

### Insomnia Measurements

The Southern University of Science and Technology Institutional Review Board approved this cross-sectional study and waived the informed consent requirement by using deidentified data. This study adheres to the Strengthening the Reporting of Observational Studies in Epidemiology (STROBE) reporting guidelines to ensure comprehensive and transparent reporting.

Insomnia in the population was estimated using daily insomnia-related social media posts in each city. Weibo, operated by Sina, is a widely used social media platform in China. According to the 2023 Weibo Annual Report, it has a daily active user base of 257 million individuals,^28^ with demographic information available in eTable 1 in Supplement 1. Traditional methods for collecting social media data were limited to posts with geotagging, overlooking a substantial portion of posts lacking independent geotagging. To address the issue, we used the internet protocol location functionality of each social media post (eFigure 1 in Supplement 1) introduced in the April 2022 release by Weibo and devised a 2-stage crawler method based on the Scrapy framework. A complete description is available in eMethods 1 in Supplement 1, and the flowchart is illustrated in eFigure 2 in Supplement 1. Briefly, in the first stage, social media posts with internet protocol location were collected on the basis of insomnia-related key words. Only social media posts containing the specified key words were included in the study. In the second stage, the publicly available personal information with location of social media users was gathered by their usernames. Because some posts containing insomnia-related key words may not truly reflect people’s insomnia, such as news or advertisements, we selected extreme gradient boosting (XGBoost) to deal with classification (eTable 2 in Supplement 1).^29,30^ See eMethods 2 in Supplement 1 for a complete description of data processing; the flowchart is illustrated in eFigure 3 in Supplement 1. The study period is defined as May 2022 to April 2023, and social media posts were summed to statistically analyze at the daily city-level scale.

To address the impact of population scale and age structure on the association between ALAN and insomnia, we used the incidence of insomnia to assess it among residents, which is defined as the number of insomnia-related social media posts per 10 000 users. To allow the data to be comparable, the user base used the population aged 15 to 39 years old in each city because this age group accounts for 96% of total social media users (eMethods 3 in Supplement 1).

### Exposure Assessment

Daily exposure levels of ALAN for each city were assessed using satellite-based ALAN intensity at 500-m spatial resolution, sourced from the National Aeronautics and Space Administration Black Marble nighttime light remote sensing images.^31^ The sensor used in this product,^31,32,33^ which exhibits substantial improvements over previous products,^33,34^ provides both visible and near-infrared light during nocturnal periods, expressed in nanowatts per square centimeter per steradian (nW/cm^2^/sr).

The gridded spatial data of ALAN images were overlaid with the city shapefile of China to extract daily average estimates of each city. We filled the missing ALAN exposure by using the mean exposure for the same day of the week for the previous and following 2 weeks. To avoid potential consequences of including outlying values of exposure data, we trimmed the highest 2.5% and lowest 2.5% of ALAN measurements in each city.^35^

### Covariates

Multiple covariates were used within the multivariable adjusted models. Daily mean meteorological data were obtained from the European Centre for Medium-Range Weather Forecasts atmospheric reanalysis of the global climate product and were processed to the city level.^36^ Temperature, relative humidity, and wind speed were selected as the potential factors influencing insomnia.^37,38,39^ The daily mean air quality index (AQI) of each city was used to adjust the impact of air pollution on insomnia, drawing data from the China National Urban Air Quality Real-time Publishing Platform. We also included weekends and holidays in the study and controlled for seasonal fixed effects.^40^

### Statistical Analysis

This is a multicity study using a time-series design. Pearson correlation coefficients were used to evaluate the association between daily ALAN exposure and the number of insomnia-related social media posts in each city. Furthermore, a multivariable adjusted linear regression was implemented to assess the association between ALAN exposure and insomnia, quantifying the increase in incidence of insomnia with each 5 nW/cm^2^/sr increment in daily mean ALAN exposure. In addition, we also examined the association in quartiles of ALAN exposure using the first quartile as a reference. The results were presented in terms of regression coefficients and corresponding 95% CIs. Four regression models were fitted, gradually adjusting for variables included in the model to evaluate the association between ALAN and insomnia. The design of model covariates is provided in eMethods 4 in Supplement 1.

To estimate the exposure-response curve between ALAN exposure and the incidence of insomnia, ALAN exposure was fit as a smoothing term in a multivariable adjusted model using a spline with 3 nodes. Subgroup analyses were conducted to examine potential effect modifications, stratified by city categories, weekend, holiday, season, temperature, and AQI separately. The subgroup analysis grouping design is provided in eMethods 4 and eFigure 4 in Supplement 1. We assessed the differences in the incidence of insomnia between different subgroups using independent samples *t* tests and Cohen *d* effect size calculation methods. Statistical significance between subgroup-specific effects was assessed using a 2-sample *z* test, and the significance threshold was defined as 0.05 between the 2 samples. All statistical tests were 2-sided, and *P* < .05 was considered statistically significant. The reported results of the incidence of insomnia represented the absolute change in percentage points.

A series of sensitivity analyses were conducted to ensure robustness of effect estimates, including adjustments for per capita gross domestic product index or social media popularity, using machine learning regression for model fitting, and conducting same process of possible insomnia-irrelevant words such as *cooking* and *dancing* to check the possibility of false-positive associations. Weibo’s popularity, which is defined by the trending score of topics discussed on the platform, was adjusted in covariates as the mean index of top 10 trending topics. The popularity index was used to control the social media post amount and the influence of public events. Machine learning regression methods included random forest, decision tree, and XGBoost. All analyses were performed using Python statistical software version 3.8.2 (Python Software Foundation) and R statistical software version 4.3.1 (R Project for Statistical Computing).

## Results

### Assessment of Exposure and Insomnia-Related Social Media Posts

Among the 336 cities in China’s mainland included in the study, 1 633 151 social media posts containing the specified key words were collected. After data processing, 1 147 583 insomnia-related social media posts were ultimately included. A complete description of the descriptive statistics for the research variables is available in eTable 3 in Supplement 1.

The spatial distribution of daily mean ALAN exposure in China at 500-m resolution is illustrated in Figure 1 from May 2022 to April 2023. Daily mean ALAN exposure during the study period across the 336 cities ranged from 3.1 to 221.0 nW/cm^2^/sr. Regionally, the distribution of ALAN exposure exhibits a pattern where the eastern regions surpass the western regions. At the urban scale, provincial capitals and economically developed urban centers exhibited higher levels of ALAN exposure. The incidence of insomnia and the number of insomnia-related social media posts also exhibited similar trends, illustrated in eFigure 5 and eFigure 6 in Supplement 1.

**Figure 1.**
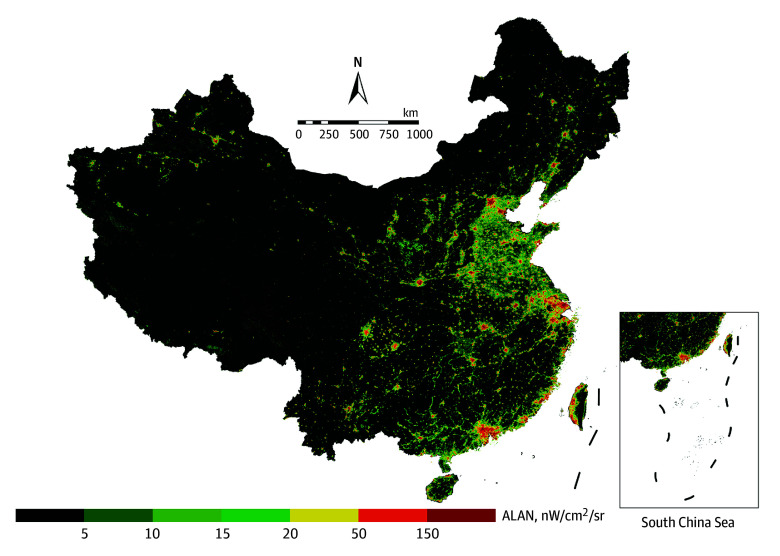
Daily Mean Spatial Distribution of Artificial Light at Night (ALAN) Exposure in China at 500-m Resolution, May 2022 to April 2023 ALAN was measured as nanowatts per centimeters squared per steradian (nW/cm^2^/sr). Inset shows islands in the South China Sea.

**Figure 2.**
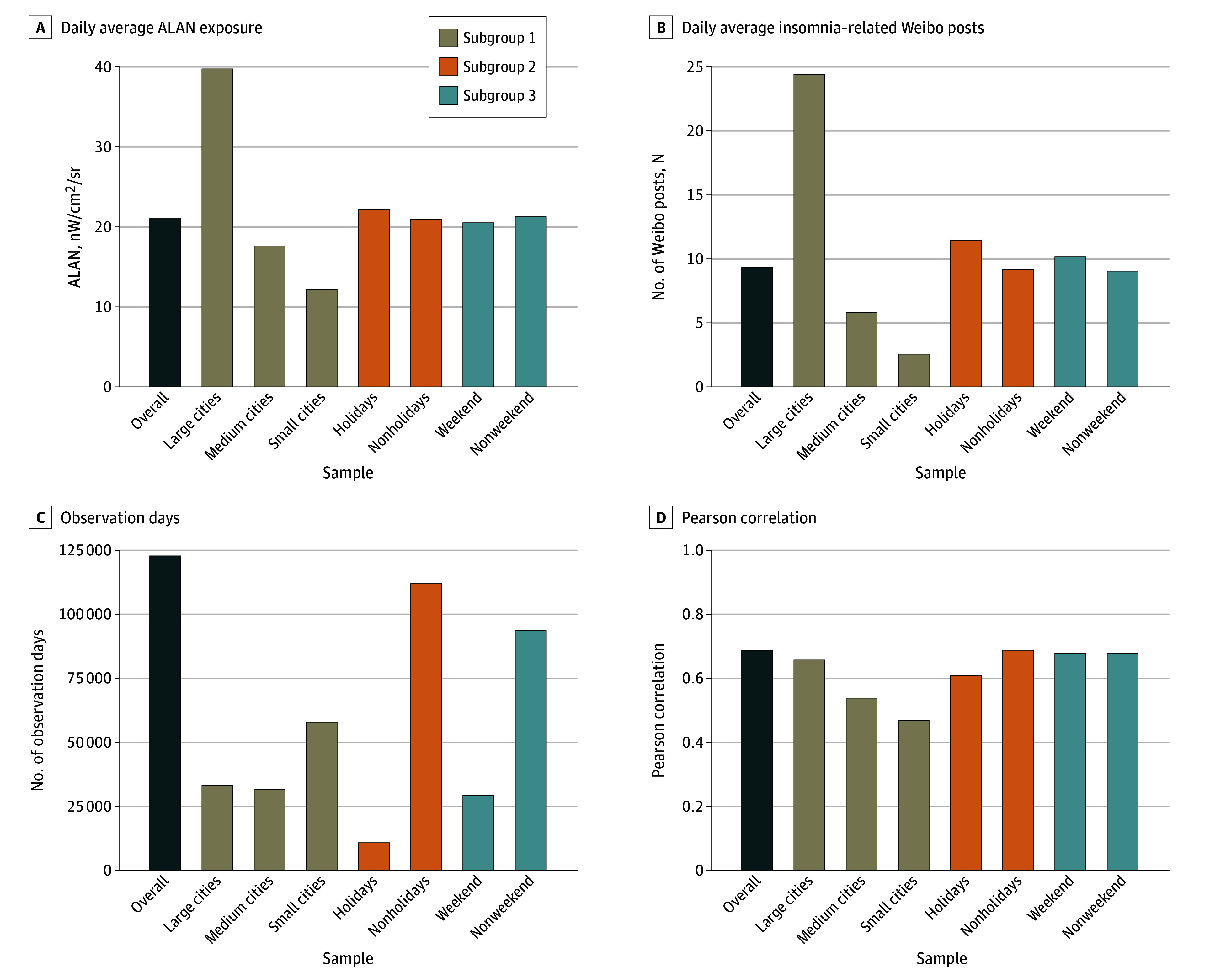
Descriptive Statistics and Pearson Correlation Coefficient of Artificial Light at Night (ALAN) and Insomnia-Related Social Media Posts A, Average ALAN exposure as nanowatts per centimeters squared per steradian (nW/cm^2^/sr) within all samples and different subgroups. B, Average daily insomnia-related social media posts in all samples and different subgroups. C, The total cumulative number of observation days in all samples and different subgroups. D, The Pearson correlation between ALAN exposure and insomnia-related social media estimations in all samples and different subgroups.

The descriptive statistics and Pearson correlation coefficients across different subgroups are presented in Figure 2 and also are detailed in eTable 4 in Supplement 1. There was a statistically significant correlation between insomnia-related posts and ALAN exposure across all groups.

### Risk Assessment

Regression results of the association between ALAN exposure and the incidence of insomnia are presented in the Table. In model 1, only considering ALAN exposure, each 5 nW/cm^2^/sr increase in ALAN corresponded to a 0.390% increase (95% CI, 0.385%-0.395%) in the incidence of insomnia. In the fully adjusted model 4, accounting for multiple covariates, the association remained, and for each 5 nW/cm^2^/sr increase in ALAN, the incidence of insomnia was estimated to increase by 0.377% (95% CI, 0.372%-0.382%). In model 4, compared with the first quartile, cities exposed to the second, third, and fourth quartile demonstrated increases of 0.569% (95% CI, 0.478%-0.661%), 1.383% (95% CI, 1.290%-1.476%), and 4.320% (95% CI, 4.208%-4.432%), respectively.

**Table.** Association Between ALAN Exposure and Insomnia

Characteristics	Quartile of ALAN exposure levels				*P* value for trend	Incidence of insomnia per 5 nW/cm^2^/sr increment in ALAN, % (95% CI)
	First	Second	Third	Fourth		
Observation days, No.^a^	30 765	30 627	30 628	30 620	NA	NA
ALAN, mean (SD), nW/cm^2^/sr	4.5 (2.6)	8.1 (5.5)	16.4 (8.6)	55.7 (48.2)	NA	NA
Daily insomnia-related posts, mean (SD), No.	3.4 (5.7)	3.9 (4.0)	5.1 (5.4)	10.0 (7.6)	NA	NA
Main models, percentage point increase in insomnia incidence (95% CI)						
Model 1^b^	0 [Reference]	0.382 (0.291**-**0.472)	1.191 (1.099**-**1.283)	4.449 (4.337**-**4.561)	<.001	0.390 (0.385**-**0.395)
Model 2^c^	0 [Reference]	0.379 (0.290**-**0.469)	1.177 (1.086**-**1.268)	4.383 (4.272**-**4.494)	<.001	0.401 (0.395**-**0.406)
Model 3^d^	0 [Reference]	1.000 (0.906**-**1.094)	1.981 (1.883**-**2.079)	5.225 (5.106**-**5.344)	<.001	0.401 (0.395**-**0.406)
Model 4^e^	0 [Reference]	0.569 (0.478**-**0.661)	1.383 (1.290**-**1.476)	4.320 (4.208**-**4.432)	<.001	0.377 (0.372**-**0.382)

Abbreviation: ALAN, artificial light at night.

^a^ Refers to the total cumulative number of observation days.

^b^ Model 1 is a crude multiple linear regression model; only ALAN was included.

^c^ Model 2 is model 1 plus adjustment for season, holiday, and weekend.

^d^ Model 3 is model 2 plus adjustment for city.

^e^ Model 4 is model 3 plus adjustment for air quality index, temperature, humidity, precipitation, and wind speed.

### Exposure-Response Curve

**Figure 3.**
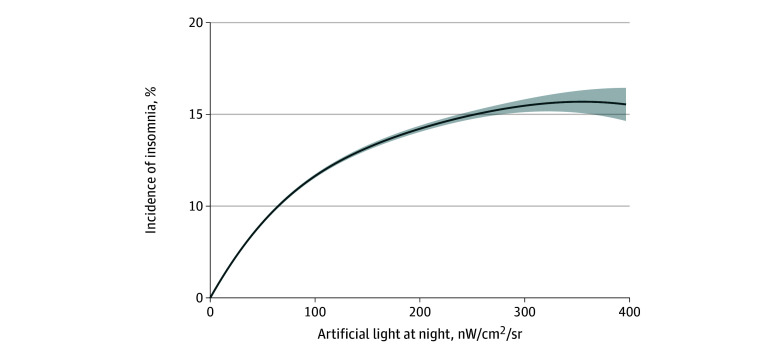
Exposure-Response Curve of Associations of Incidence of Insomnia With Artificial Light at Night Exposure To estimate the exposure-response curves between artificial light at night exposure as nanowatts per centimeters squared per steradian (nW/cm^2^/sr) and the incidence of insomnia, exposure was fit as a smoothing term in a multivariable adjusted model using a spline with 3 nodes. Shaded area denotes 95% CI. The exposure of daily artifical light at night at the city level ranged from 0 to 396.55 nW/cm^2^/sr among all days in the study period.

Fitted exposure-response function of the incidence of insomnia within the multivariable adjusted models is illustrated in Figure 3, demonstrating an upward trend. The association between ALAN exposure and the incidence of insomnia was nonlinear throughout the entire exposure range, which was relatively steep at low ALAN exposures and leveled off at higher ALAN exposures. It is noteworthy that the daily mean ALAN exposure in nearly two-thirds of Chinese cities ranged from 10 to 80 nW/cm^2^/sr, falling within the scope of rapid growth.

### Subgroup Analysis

**Figure 4.**
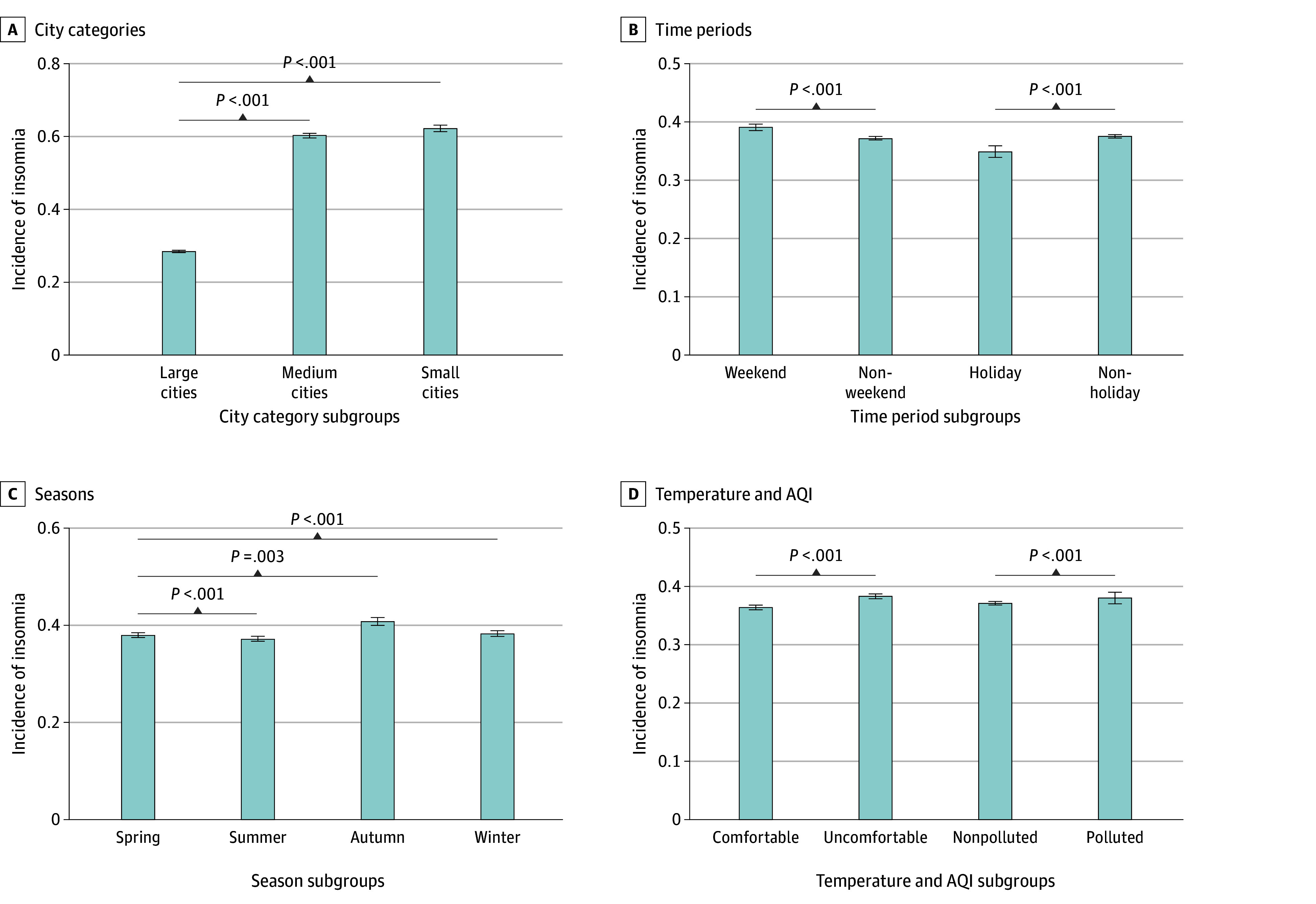
Subgroup Analyses of the Association Between Insomnia Incidence and Each 5 nW/cm^2^/sr Increase in Artificial Light at Night Exposure A, Results of subgroup analysis by different city categories, divided into large cities (population >5 million), medium cities (population ≥3 million to ≤5 million), and small cities (population <3 million). B, Results of subgroup analysis by different time periods, where weekends and holidays were determined on the basis of the notices issued by the General Office of the State Council regarding holiday arrangements. C, Results of subgroup analysis by seasons, divided into spring (March to May), summer (June to August), autumn (September to November), and winter (December to February of the following year). D, Results of subgroup analysis by different temperature conditions and air quality. Temperature stratification was based on the specific temperature distribution for each city, with the range between the 25th and 75th percentiles defined as comfortable temperature, whereas the remainder was categorized as uncomfortable temperature. Days were classified as polluted when the air quality index (AQI) exceeded 101 and as nonpolluted when it was 100 or below.

The regression coefficients and 95% CIs of the association between ALAN exposure and the incidence of insomnia across different subgroups are provided in Figure 4. Sample estimate and measures of differences are provided in eTable 5 in Supplement 1. Overall, the health outcomes of ALAN exposure on the incidence of insomnia were statistically significant among the separate subgroups. Compared with large cities, the estimated impact of ALAN exposure on insomnia was significantly higher in medium cities and small cities. Specifically, for each 5 nW/cm^2^/sr increase in ALAN exposure, the incidence of insomnia in medium cities and small cities was estimated to increase by 0.603% (95% CI, 0.591%-0.615%) and 0.622% (95% CI, 0.605%-0.639%), respectively, whereas the increase in large cities was only 0.284% (95% CI, 0.278%-0.289%). Furthermore, the estimated impact of ALAN exposure on insomnia varied across different time periods and seasons. The effect also increased when temperature conditions fell within the lowest and highest quartiles, as well as during periods of poor air quality (AQI ≥101).

### Sensitivity Analysis

We found similar results when we additionally adjusted the main models for per capita gross domestic product or social media popularity and observed similar estimates when using machine learning regression for model fitting. Posts related to other irrelevant words did not show a false-positive association with ALAN exposure. All sensitivity analyses are illustrated in eTable 6 and eFigure 7 in Supplement 1.

## Discussion

This cross-sectional ecological study was based on large-scale social media data from a rapidly expanding ALAN exposure country to comprehensively investigate the association between ALAN exposure and population insomnia, involving 1 147 583 insomnia-related social media posts from 336 cities in China over the course of the period from May 2022 to April 2023. The study results provide evidence of the association between increased ALAN exposure and a higher incidence of insomnia.

Several biological mechanisms have been proposed to explain the association between ALAN exposure and insomnia, including dysregulation of pineal gland function^8,41^ and melatonin secretion,^9,42^ altered neuronal circuits,^43^ and activation of oxidative stress genes.^44^ Melatonin, a hormone produced by the pineal gland, plays a crucial role in regulating sleep-wake cycles and other biological rhythms.^42^ High ALAN exposure can interfere with pineal gland function, inhibit melatonin secretion, disrupt circadian rhythm,^41^ and contribute to insomnia. Some studies^44,45^ have also suggested that long-term exposure to ALAN can activate oxidative stress genes, impacting human cell function and circadian rhythm.

Current research on the association between ALAN exposure and insomnia mainly comes from developed countries such as Europe, the US, and Japan.^8,18,46,47,48^ Findings from these studies consistently indicate that elevated ALAN exposure heightens the risk of insomnia. Although we found similar outcomes, noteworthy distinctions exist. Different from the patterns observed in developed countries, the association between ALAN exposure and insomnia in our study was higher in medium and small cities compared with large cities. This divergence may be attributed to disparities in ALAN distribution, regional development stages, and urban lighting planning.^3^ The uneven distribution of ALAN exposure in China (Figure 1), coupled with substantial variations in economic levels between cities, contributes to distinct outcomes. More importantly, ALAN exposure levels may be low in certain cities in the early stages of development. However, the absence of pertinent policies and standards in the city development^49,50,51^ could result in inadequately designed lighting facilities and urban planning, leading to excessive or scattered light,^51^ consequently greatly elevating the risk of insomnia among the residents in small and medium cities. Furthermore, social media platforms represented by Weibo can provide a more comprehensive and unfiltered perspective on insomnia, incorporating into the study those experiencing insomnia in small-to-medium cities who may otherwise be overlooked. In summary, our findings indicate that ALAN exposure poses a threat to insomnia, with potentially higher risks in small and medium cities where ALAN levels are relatively lower.

Social media serves as a valuable resource for capturing real-time living conditions and emotional changes among a vast and unexplored population, providing an extensive, unfiltered perspective on the daily lives of residents. Among previous research in China, Weibo stands out as the most popular platform for assessing health effects. Previous studies have used this platform to explore the association between different factors and the population happiness index, such as fine particulate matter exposure^25,27^ and temperature changes.^52^ Our study distinguishes itself from previous research on insomnia factors that used Weibo^53^ by offering a clearer focus on ALAN exposure and better adjusting for regional demographic factors. Using a 2-stage crawler method, we incorporated insomnia-related social media posts lacking independent geotagging, which enables us to explore the association between ALAN and insomnia at the city level and on a daily scale in a more comprehensive base.

To the best of our knowledge, nationwide studies in China’s mainland investigating the association between ALAN exposure and the incidence of insomnia are still limited. A previous study^21^ reported the association between ALAN exposure and sleep disorders among children in 3 provinces of China, using questionnaire surveys on children’s sleep patterns and exposure assessment based on satellite-based night light images. Two other studies^6,20^ focused on elderly and retired military people, respectively, focusing on sleep quality and duration rather than the incidence of insomnia. By using large-scale, up-to-date social media data and ALAN exposure models with fine spatial resolution, we were able to document variations in the incidence of insomnia across the study population nationwide. Our findings suggest that ALAN may be an important risk factor associated with insomnia, which may guide further research into the potential role of ALAN exposure in sleep issues as a determinant of health disparities. Furthermore, this study emphasizes the importance of rational planning and layout of artificial nighttime lighting installations, particularly in small-to-medium cities. Currently, developing countries such as China still lack sufficient outdoor lighting standards and regulations. Thus, implementing local light pollution prevention policies is needed to protect population health benefit along with economic development.

### Limitations

Some limitations of our study should be noted. First, no information was collected on the extent and duration of indoor ALAN exposure, which may cause bias in estimates of sleep quality effects. Second, some confounding factors such as screen exposure and outdoor noise were not considered in the study owing to a lack of precise data sources. Third, despite using an innovative social media data collection method, our study was limited to the city level and could not further investigate individual health effects. In addition, the exclusion of nonpublic insomnia-related posts likely led to an underestimation of the actual impact of ALAN exposure. Furthermore, owing to the unavailability of regional social media user proportions, we used the population aged 15 to 39 years old as a proxy for social media users in each city, which may not fully capture regional differences in social media usage. Our future work will consider new data sources and update the collection method to uncover the population’s insomnia more precisely.

## Conclusions

This study provides evidence demonstrating that high ALAN exposure is a factor associated with substantial risk of insomnia in China. These findings contribute to the understanding of the detrimental health effects associated with intense nighttime light pollution. Moreover, the results emphasize the potential benefits to population health that can be achieved by implementing thoughtful planning of artificial lighting during the early stages of development.

## Notes

Supplement 1. **eMethods 1.** Complete Description of "Two-Stage Crawler" Methodology Based on Scrapy to Extract Insomnia-related Weibo Posts **eMethods 2.** Complete Description of Insomnia-related Weibo Posts Processing **eMethods 3.** Definition of the Incidence of Insomnia **eMethods 4.** Grouping Criteria for City Categories, Time Factors and Climatic Conditions **eTable 1.** Age and Sex Distribution of Weibo Users in 2020 **eTable 2.** Evaluation Results of Different Machine Learning Models for Filtering News, Advertisements, and Texts from Users Actively Staying Up Late **eTable 3.** Descriptive Summary of Daily Insomnia Measurements, ALAN Exposure and Covariates at The City Level **eTable 4.** Descriptive Summary and Pearson Correlation Coefficient of ALAN Exposure and Insomnia-related Weibo Posts **eTable 5.** Differences Analyses of Associations Between ALAN Exposure and Incidence of Insomnia in Subgroups **eTable 6.** Sensitivity Analyses and False Positive Assessment of Associations Between ALAN Exposure and Incidence of Insomnia **eFigure 1.** Example of Insomnia-related Weibo Post with IP Location Along with Post and Shown in User Homepage **eFigure 2.** Flowchart of The ‘Two-Stage Crawler’ Methodology in The Extraction Process of Weibo Posts **eFigure 3.** Complete Description of Insomnia-related Weibo Posts Processing in Weibo Classification Process **eFigure 4.** Categorization of Cities Based on Population Size in China **eFigure 5.** Distribution of The Monthly Average Number of Insomnia-related Weibo Posts at The City Level in China **eFigure 6.** Distribution of Average Daily Incidence of Insomnia at The City Level in China **eFigure 7.** The Results of The Top Eight Feature Importance Rankings for Three Machine Learning Models

Supplement 2.
Data Sharing Statement
