# Supplementary material for: Outdoor Artificial Light at Night and Insomnia-Related Social Media Posts
Source: JAMA Netw Open. 2024 Nov 20;7(11):e2446156. doi: 10.1001/jamanetworkopen.2024.46156 (PMC11579793; doi:10.1001/jamanetworkopen.2024.46156)
Supplement: Supplement 1. — eMethods 1. Complete Description of "Two-Stage Crawler" Methodology Based on Scrapy to Extract Insomnia-related Weibo Posts eMethods 2. Complete Description of Insomnia-related Weibo Posts Processing eMethods 3. Definition of the Incidence of Insomnia eMethods 4. Grouping Criteria for City Categories, Time Factors and Climatic Conditions eTable 1. Age and Sex Distribution of Weibo Users in 2020 eTable 2. Evaluation Results of Different Machine Learning Models for Filtering News, Advertisements, and Texts from Users Actively Staying Up Late eTable 3. Descriptive Summary of Daily Insomnia Measurements, ALAN Exposure and Covariates at The City Level eTable 4. Descriptive Summary and Pearson Correlation Coefficient of ALAN Exposure and Insomnia-related Weibo Posts eTable 5. Differences Analyses of Associations Between ALAN Exposure and Incidence of Insomnia in Subgroups eTable 6. Sensitivity Analyses and False Positive Assessment of Associations Between ALAN Exposure and Incidence of Insomnia eFigure 1. Example of Insomnia-related Weibo Post with IP Location Along with Post and Shown in User Homepage eFigure 2. Flowchart of The ‘Two-Stage Crawler’ Methodology in The Extraction Process of Weibo Posts eFigure 3. Complete Description of Insomnia-related Weibo Posts Processing in Weibo Classification Process eFigure 4. Categorization of Cities Based on Population Size in China eFigure 5. Distribution of The Monthly Average Number of Insomnia-related Weibo Posts at The City Level in China eFigure 6. Distribution of Average Daily Incidence of Insomnia at The City Level in China eFigure 7. The Results of The Top Eight Feature Importance Rankings for Three Machine Learning Models [file jamanetwopen-e2446156-s001.pdf]

Supplemental Online Content

Duan J, Li Q, Yin Z, et al. Outdoor artificial light at night and insomnia-related social media posts. *JAMA Netw Open*. 2024;7(11):e2446156. doi:10.1001/jamanetworkopen.2024.46156

eMethods 1. Complete Description of "Two-Stage Crawler" Methodology Based on Scrapy to Extract Insomnia-related Weibo Posts
eMethods 2. Complete Description of Insomnia-related Weibo Posts Processing
eMethods 3. Definition of the Incidence of Insomnia
eMethods 4. Grouping Criteria for City Categories, Time Factors and Climatic Conditions
eTable 1. Age and Sex Distribution of Weibo Users in 2020
eTable 2. Evaluation Results of Different Machine Learning Models for Filtering News, Advertisements, and Texts from Users Actively Staying Up Late
eTable 3. Descriptive Summary of Daily Insomnia Measurements, ALAN Exposure and Covariates at The City Level
eTable 4. Descriptive Summary and Pearson Correlation Coefficient of ALAN Exposure and Insomnia-related Weibo Posts
eTable 5. Differences Analyses of Associations Between ALAN Exposure and Incidence of Insomnia in Subgroups
eTable 6. Sensitivity Analyses and False Positive Assessment of Associations Between ALAN Exposure and Incidence of Insomnia
eFigure 1. Example of Insomnia-related Weibo Post with IP Location Along with Post and Shown in User Homepage
eFigure 2. Flowchart of The 'Two-Stage Crawler' Methodology in The Extraction Process of Weibo Posts
eFigure 3. Complete Description of Insomnia-related Weibo Posts Processing in Weibo Classification Process
eFigure 4. Categorization of Cities Based on Population Size in China
eFigure 5. Distribution of The Monthly Average Number of Insomnia-related Weibo Posts at The City Level in China
eFigure 6. Distribution of Average Daily Incidence of Insomnia at The City Level in China
eFigure 7. The Results of The Top Eight Feature Importance Rankings for Three Machine Learning Models

This supplemental material has been provided by the authors to give readers additional information about their work.

eMethods 1. Complete Description of "Two-Stage Crawler" Methodology Based on Scrapy to Extract Insomnia-related Weibo Posts

Traditional methods for collecting Weibo texts generally used two approaches: (1) utilizing advanced Weibo search to specify the geographical location to a particular city, crawling all geotagged Weibo posts within that city, and conducting keyword extraction and processing; (2) gathering Weibo data with geotags over a specific time period and allocating Weibo posts to the research area based on latitude and longitude. Both methods exclusively select geotagged Weibo posts for analysis, disregarding a significant amount of content without geotags.

To address this issue, a two-stage crawling methodology is applied for data collection, see **eFigure 2**. In the first stage, Weibo content collection is based on keywords, the following terms: insomnia ("失眠"), sleeplessness ("睡不着"), difficulty falling asleep ("难以入睡"), tossing and turning ("辗转反侧"), poor sleep quality ("睡眠差"), unable to sleep ("没睡着"). As IP addresses only display provincial-level administrative regions, they cannot be pinpointed to the city level administrative regions. Therefore, in the second stage, the reported location displayed on the homepage of the user who published Weibo was collected.

eMethods 2. Complete Description of Insomnia-related Weibo Posts Processing

The processing of Sina Weibo data is divided into two steps: geographical comparison of Weibo posts and text classification based on machine learning algorithms.

The first step is geographical comparison. Using a "two-stage crawler," we collected both the IP location and the user-reported location of Weibo users. For each Weibo post, if the IP location and the user-reported location are inconsistent, the data is excluded. This strategy aims to enhance the accuracy of the user's city location, thereby minimizing the potential impact of location bias on the analysis results. The second step is text classification based on machine learning algorithms, which includes three phases: data preprocessing and feature extraction, model training and hyperparameter optimization, and test set evaluation.

We preprocessed the raw text data to ensure its quality and consistency. The preprocessing steps included removing non-Chinese text, tokenization, removing stopwords, and other text-cleaning tasks. Next, feature extraction was performed on the Sina Weibo data using the TfidfVectorizer (TF-IDF) algorithm to generate word vectors. This process returned feature vectors and labels for each set, as well as feature names. We set the maximum number of features to 15,000 and considered n-gram features ranging from unigrams to trigrams. Due to the lack of available open datasets, we created separate training sets (each consisting of 5,000 instances) and test sets (each consisting of 1,000 instances) for news, advertisements, and active staying-up texts. Three researchers, experienced in text classification, independently and concurrently annotated the datasets. In cases where there was a disagreement on the labels, the majority decision principle was applied. Based on previous studies, extreme gradient boosting (XGBoost), Random Forest (RF), and Support Vector Machine (SVM) were identified as possible approaches for text classification, so we trained the models using these three machine learning algorithms. We employed three-fold cross-validation for hyperparameter selection and optimization. Grid search was used to test different combinations of hyperparameters on the training data to identify the best model configuration. Since internal validation within the training set cannot demonstrate the model's stability and generalizability, we conducted final model evaluation on an independent test set after completing the model training and hyperparameter optimization. By using the optimal hyperparameter configuration determined during the training phase, we made predictions on the test set and calculated precision, recall, F1 score (i.e. a metric that balances precision and recall in classification tasks, representing the harmonic mean of precision and recall), and accuracy. In selecting different models, avoiding "Type II errors", where news, advertisements, and active staying-up texts are misclassified as insomnia-related texts, was a priority. Therefore, recall was set as the primary evaluation metric, while other metrics were also considered in a comprehensive assessment. The precision, recall, F1 score, and accuracy for each machine learning model on the test set are shown in the **eTable 2**.

The results indicate that XGBoost, a machine learning algorithm well-suited for handling large-scale features and with strong generalization capabilities, achieved the best classification performance on the test set. As a result, we selected the XGBoost algorithm for the text classification task on Weibo data, filtering out news, advertisements, and active staying-up posts. Ultimately, 1,147,583 insomnia-related Weibo posts were included in the study. A detailed description of the processing of insomnia-related posts is provided in **eFigure 3**.

© 2024 Duan J et al. *JAMA Network Open.*

eMethods 3. Definition of the Incidence of Insomnia

Traditional research using Sina Weibo for gauging collective emotional intensity typically applies the quantity of keyword-related Weibo posts as the dependent variable. However, due to variations in population size among different cities, the absolute number of such posts inadequately encapsulates the total number of insomnia cases within each city. In order to incorporate demographic factors into the dependent variable design, we introduced the 'Incidence of Insomnia' index to evaluate the distribution of insomnia across diverse regions. To mitigate the influence of local population structure on the dependent variable, we set the user amount as the population aged 15 to 39 years old which distributed in 96% of all Weibo users. Ultimately, the incidence of insomnia is characterized by the number of insomnia-related Weibo posts among every 10,000 Weibo users. The formula for this rate is as follows:

$$I_{ci} = \frac{N_{ci} \times 10000}{P_c \times a_c} \quad (1)$$

In the formula: I_{ci} represents the incidence of insomnia in each city on the i-th day. N_{ci} signifies the number of insomnia-related Weibo posts in a given city on the i-th day; P_c denotes the urban population; a_c represents the Weibo user coefficient, defined as the proportion of the population aged 15 to 39 years old, with data sourced from China's Seventh National Census; and c spans from 1 to 336.

© 2024 Duan J et al. *JAMA Network Open.*

eMethods 4. Grouping Criteria for City Categories, Time Factors and Climatic Conditions

Covariates incorporated in the models comprised daily mean temperature, relative humidity, wind speed, air quality index (AQI), city categories, time factors, and seasons. We categorized 336 cities in China into three tiers based on their permanent population size: Large Cities, Medium Cities, and Small Cities. Large Cities are defined as cities with a population of over 5 million, Medium Cities as those with a population between 3 and 5 million, and cities with a population below 3 million are categorized as Small Cities. **eFigure 5** presents a detailed categorization of cities based on population size. Weekends and holidays were determined according to the notices issued by the General Office of the State Council regarding holiday arrangements. The seasons were defined as follows: spring from March to May, summer from June to August, autumn from September to November, and winter from December to February of the following year. Temperature stratification was based on the temperature distribution specific to each city, where the range between the 25th and 75th percentiles was defined as "comfortable temperature," and the remainder as "uncomfortable temperature." Days were categorized as polluted days when the AQI exceeded 101 and non-polluted days when it was 100 or below.

© 2024 Duan J et al. *JAMA Network Open.*

eTable 1. Age and Sex Distribution of Weibo Users in 2020

Age Group [a]	Percentage	Women (%)	Men (%)
**1960s or earlier**	1%	41.1	58.9
**1970s**	3%	45.1	54.9
**1980s**	18%	47.7	52.3
**1990s**	48%	54.1	45.9
**2000s**	30%	61.6	38.4
**Overall**	-	54.6	45.4

[a] Data sourced from the latest Weibo Users Report in 2020[1], where 1960s or earlier, 1970s, 1980s, 1990s, and 2000s refer to people born in 1960 to 1969 or earlier, 1970 to 1979, 1980 to 1989, 1990 to 1999, and 2000 to 2009 or later, respectively.

1. Weibo report publishing platform-Micro report. Accessed January 12, 2024. https://data.weibo.com/report/index

© 2024 Duan J et al. *JAMA Network Open.*

eTable 2. Evaluation Results of Different Machine Learning Models for Filtering News, Advertisements, and Texts from Users Actively Staying Up Late

Type	Metric [a]	XGBoost	Random Forest	SVM
News	Precision	0.834	0.759	0.813
	Recall	0.918	0.84	0.858
	F1	0.874	0.797	0.835
	Accuracy	0.894	0.829	0.864
Advertisements	Precision	0.873	0.766	0.853
	Recall	0.810	0.303	0.795
	F1	0.841	0.434	0.823
	Accuracy	0.877	0.684	0.863
Texts of Users Actively Staying up Late	Precision	0.824	0.841	0.805
	Recall	0.833	0.753	0.828
	F1	0.828	0.794	0.816
	Accuracy	0.862	0.844	0.851

XGBoost, extreme gradient boosting; SVM, support vector machine.

[a] Multiple estimation metrics to assess model, where Precision is the proportion of true positives among predicted positives, Recall is the proportion of true positives among actual positives, F1 score is the harmonic mean of Precision and Recall, and Accuracy is the proportion of true positives and true negatives among all instances.

© 2024 Duan J et al. *JAMA Network Open.*

eTable 3. Descriptive Summary of Daily Insomnia Measurements, ALAN Exposure and Covariates at The City Level

Variable	Mean (SD)	Median (IQR)	Q_1	Q_{99}
Insomnia-related Weibo posts (N) [a]	9.4 ± 18.2	4.0 (7)	0	83
Incidence of Insomnia (%)	5.6 ± 6.4	3.9 (6.8)	0	29.3
ALAN (nW/cm^2/sr)	21.2 ± 32.0	10.2 (18.3)	0.8	162.9
Temperature (°C)	18.2 ± 10.7	18.2 (15.0)	-13.4	35.0
Humidity (%)	64.3 ± 19.1	67.0 (29.4)	19.7	95.4
Air Quality Index	57.8 ± 43.3	47.3 (34.8)	15.5	235.8

ALAN, artificial light at night; SD, standard deviation; IQR, interquartile range; Q_1, 1st percentile; Q_{99}, 99th percentile.

[a] The number of daily average insomnia-related Weibo posts.

© 2024 Duan J et al. *JAMA Network Open.*

eTable 4. Descriptive Summary and Pearson Correlation Coefficient of ALAN Exposure and Insomnia-related Weibo Posts

Variable	Observation days [a]	Weibo posts (N) [b]	ALAN ($nW/cm^2/sr$)	Pearson
**Overall**	122,640	9.4 ± 18.2	21.2 ± 32.0	0.69*
**City** [c]				
**Large Cities**	33,215	24.4 ± 28.7	39.9 ± 48.9	0.66*
**Medium Cities**	31,755	5.8 ± 7.9	17.7 ± 20.7	0.54*
**Small Cities**	57,670	2.6 ± 4.0	12.3 ± 16.6	0.47*
**Time** [d]				
**Holidays**	10,752	11.5 ± 21.3	22.2 ± 33.5	0.61*
**Non-Holidays**	111,888	9.2 ± 17.9	21.1 ± 31.9	0.69*
**Weekend**	29,232	10.2 ± 17.8	20.7 ± 31.6	0.68*
**Non-Weekend**	93,408	9.1 ± 19.5	21.3 ± 32.1	0.68*

ALAN, artificial light at night; Pearson, Pearson correlation coefficient; N, the cumulative count.

[a] The total cumulative number of observation days.

[b] The number of daily average insomnia-related Weibo posts.

[c] City categorization: Large Cities (population > 5 million), Medium Cities (3 million ≤ population ≤ 5 million), and Small Cities (population < 3 million).

[d] Time: Weekends and holidays were determined according to the notices issued by the General Office of the State Council regarding holiday arrangements

eTable 5. Differences Analyses of Associations Between ALAN Exposure and Incidence of Insomnia in Subgroups

Variable	Observation days [a]	The incidence of insomnia (%)	Difference [b]	*P*-value [c]	Cohen's d effect size [d] (95%CI)
**Overall**	122,640	5.6 (6.4)			
**City [e]**					
**Large Cities**	33,215	7.2 (5.9)		Reference	
**Medium Cities**	31,755	4.7 (5.2)	2.5	< 0.001	0.46 (0.37-0.54)
**Small Cities**	57,670	5.2 (7.1)	2	< 0.001	0.31 (0.22-0.40)
**Holiday [f]**					
**Holidays**	10,752	7.2 (7.6)		Reference	
**Non-Holidays**	111,888	5.4 (6.2)	1.8	< 0.001	0.27 (0.14-0.40)
**Weekend [f]**					
**Weekends**	29,232	6.2 (6.8)		Reference	
**Non-Weekends**	93,408	5.4 (6.3)	0.8	< 0.001	0.13 (0.05-0.21)
**Season [g]**					
**Spring**	30,912	6.0 (6.7)		Reference	
**Summer**	30,912	5.0 (5.9)	1	< 0.001	0.16 (0.06-0.26)
**Autumn**	30,576	5.8 (6.5)	0.2	0.003	0.03 (-0.08-0.13)
**Winter**	30,240	5.6 (6.4)	0.4	< 0.001	0.06 (-0.04-0.16)
**Temperature [h]**					
**Comfortable**	61,152	5.8 (6.5)		Reference	
**Uncomfortable**	61,488	5.4 (6.3)	0.4	< 0.001	0.06 (-0.02-0.13)
**AQI [i]**					
**Non-polluted**	111,219	5.7 (6.5)		Reference	
**Polluted**	11,421	4.7 (5.5)	1	< 0.001	0.16 (0.03-0.28)

ALAN, artificial light at night; CI, confidence interval.

[a] The total cumulative number of observation days.

[b] The difference of mean values within different groups.

[d] Effect sizes are metrics used Cohen's d effect size to quantify the strength or magnitude of differences between two groups. assessing the practical significance of results.

[c] *P*-value was calculated by a 2-sample z-test and the significance threshold was defined as 0.05 between the 2 samples and Bonferroni calibration result for multiple samples (0.017 for 3 samples).

[e] City categorization: Large Cities (population > 5 million), Medium Cities (3 million ≤ population ≤ 5 million), and Small Cities (population < 3 million).

[f] Holidays and Weekends were determined according to the notices issued by the General Office of the State Council regarding holiday arrangements.

[g] Spring (March to May), Summer (June to August), Autumn (September to November), Winter (December to February of the following year).

[h] Temperature stratification was based on the temperature distribution specific to each city, where the range between the 25th and 75th percentiles was defined as "comfortable temperature," and the remainder as "uncomfortable temperature."

[i] Categorized as polluted days when the AQI exceeded 101 and non-polluted days when it was 100 or below.

© 2024 Duan J et al. *JAMA Network Open.*

eTable 6. Sensitivity Analyses and False Positive Assessment of Associations Between ALAN Exposure and Incidence of Insomnia

Sensitivity Analyses	ALAN, 5 nW/cm²/sr Coefficient (95% CI)	*P*-value
**Main Analysis** [a]		
Main Model	0.377% (0.372%-0.382%)	*<0.001*
**Sensitivity Analysis** [b]		
Main Model + GDP	0.143% (0.137%-0.148%)	*<0.001*
Main Model + Weibo Popularity	0.378% (0.372%-0.383%)	*<0.001*
**False Positive Testing** [c]		
'Cooking'	-0.002% (-0.005%-0.002%)	*0.37*
'Dancing'	-0.003% (-0.007%-0.002%)	*0.21*

ALAN, artificial light at night; GDP, gross domestic product.

[a] Main model is multiple linear regression model adjusted for season, holiday, weekend, city size, air quality, and meteorological factors.

[b] Sensitivity analysis conducted based on main model with further adjustment for GDP and Weibo popularity defined by the mean value of top ten most discussed topic in Weibo.

[c] False positive testing conducted in the same process of main analysis, with different irrelated word.

© 2024 Duan J et al. *JAMA Network Open.*

eFigure 1. Example of Insomnia-related Weibo Post with IP Location Along with Post and Shown in User Homepage

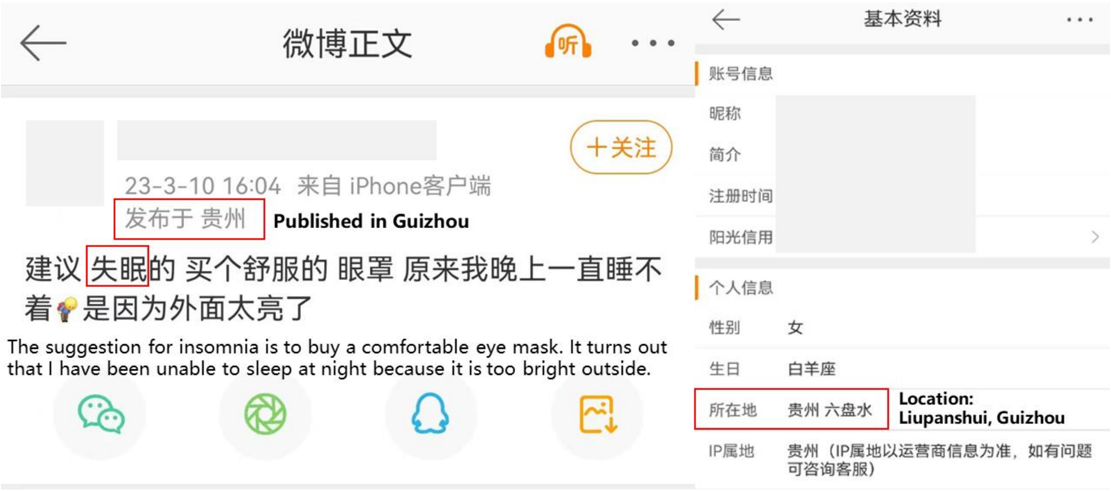

All the contents were deidentified to remain the privacy of users.

© 2024 Duan J et al. *JAMA Network Open.*

eFigure 2. Flowchart of The ‘Two-Stage Crawler’ Methodology in The Extraction Process of Weibo Posts

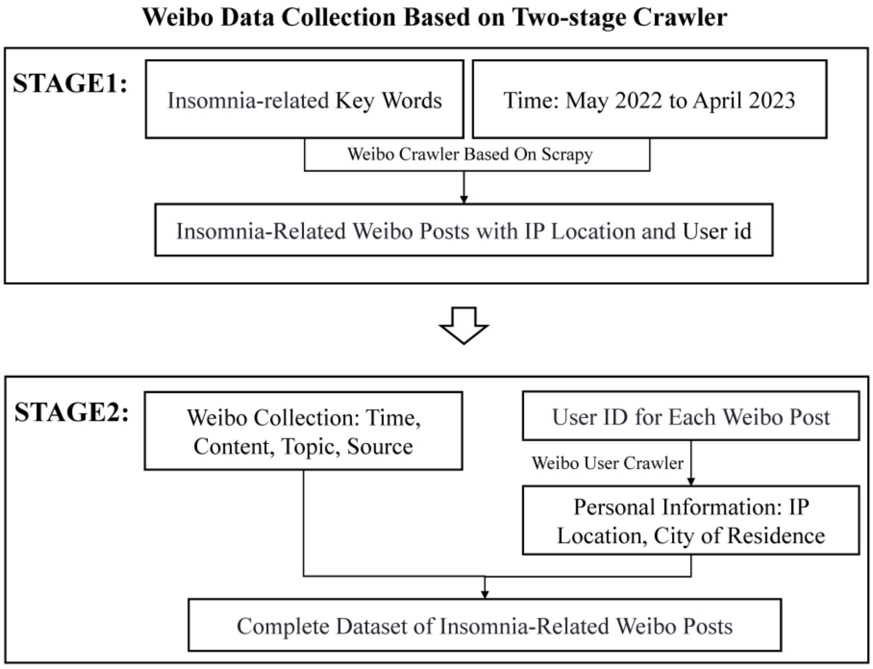

© 2024 Duan J et al. *JAMA Network Open.*

eFigure 3. Complete Description of Insomnia-related Weibo Posts Processing in Weibo Classification Process

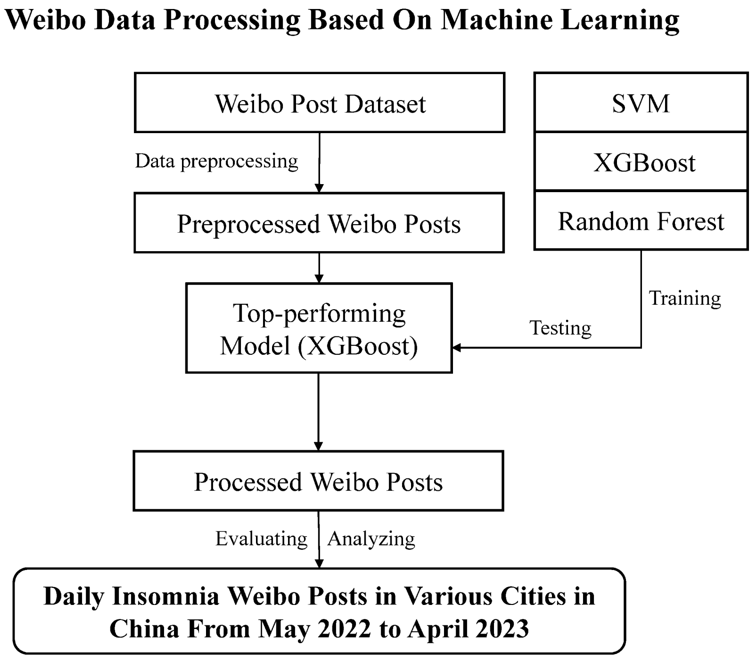

SVM: support vector machine; XGBoost: extreme gradient boosting; Random Forest: random forest algorithm

© 2024 Duan J et al. *JAMA Network Open.*

eFigure 4. Categorization of Cities Based on Population Size in China

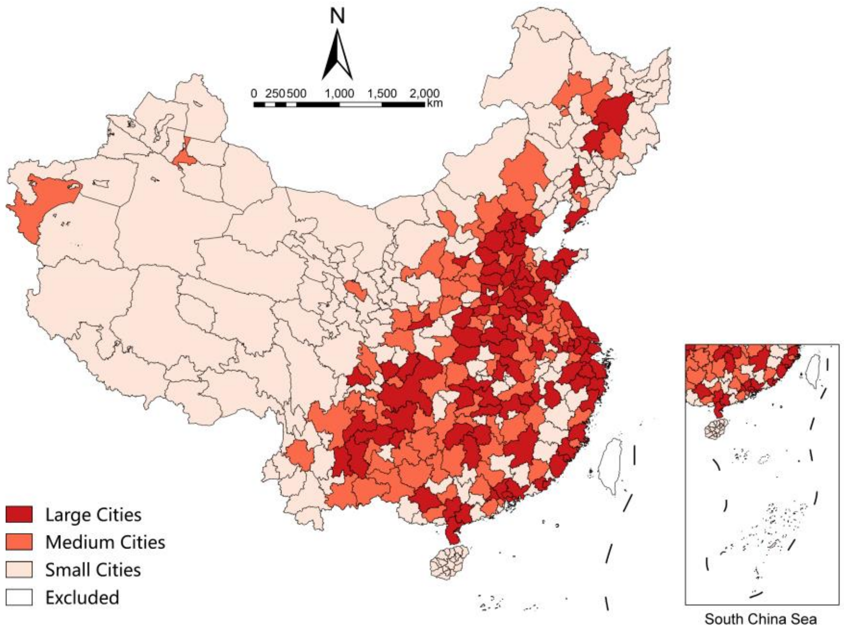

City categorization: Large Cities (population > 5 million), Medium Cities (3 million ≤ population ≤ 5 million), and Small Cities (population < 3 million).

© 2024 Duan J et al. *JAMA Network Open.*

eFigure 5. Distribution of The Monthly Average Number of Insomnia-related Weibo Posts at The City Level in China

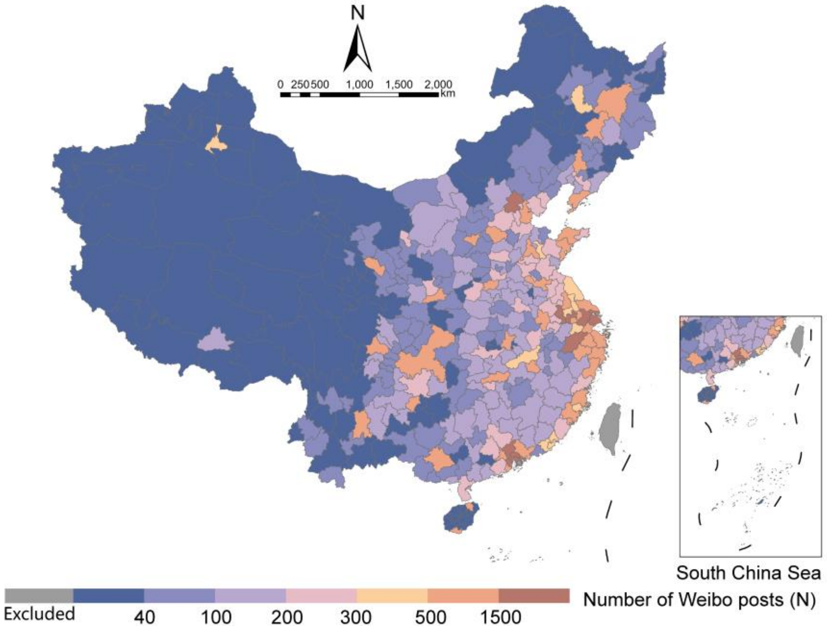

© 2024 Duan J et al. *JAMA Network Open.*

eFigure 6. Distribution of Average Daily Incidence of Insomnia at The City Level in China

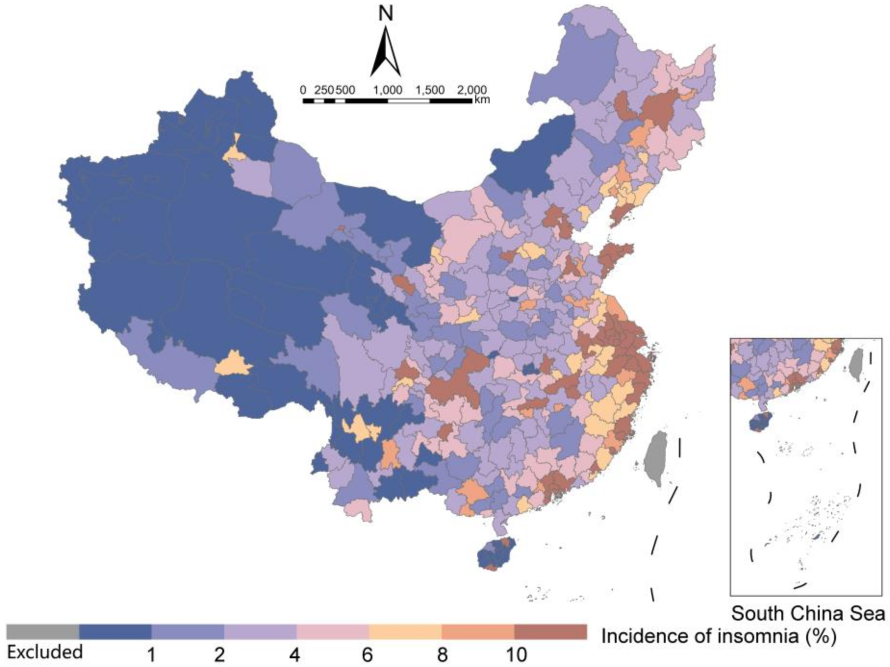

© 2024 Duan J et al. *JAMA Network Open.*

eFigure 7. The Results of The Top Eight Feature Importance Rankings for Three Machine Learning Models

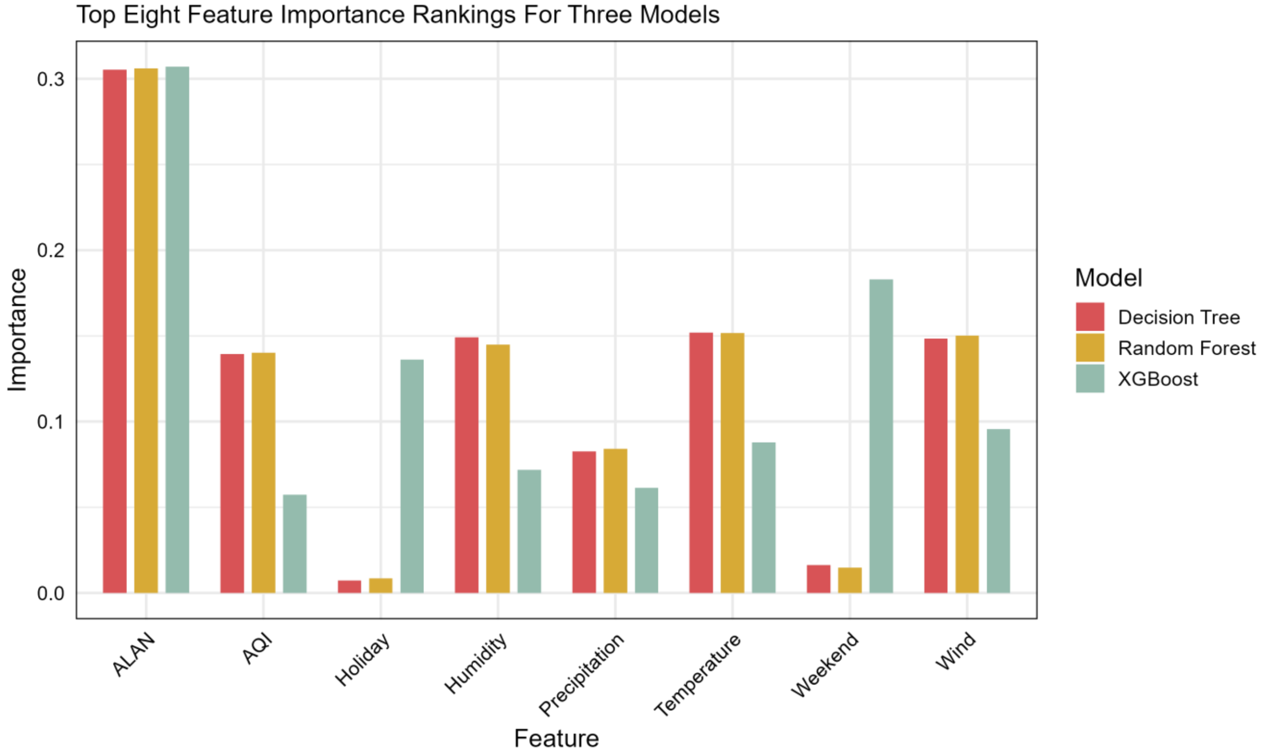

ALAN: artificial light at night (nW/cm²/sr); AQI: Air Quality Index; XGBoost, extreme gradient boosting

© 2024 Duan J et al. *JAMA Network Open.*
