# Supplementary material for: Outdoor Artificial Light at Night and Insomnia-Related Social Media Posts
Source: JAMA Netw Open. 2024 Nov 20;7(11):e2446156. doi: 10.1001/jamanetworkopen.2024.46156 (PMC11579793; doi:10.1001/jamanetworkopen.2024.46156)
Supplement: Supplement 2. — Data Sharing Statement [file jamanetwopen-e2446156-s002.pdf]

Data Sharing Statement

Duan. Outdoor Artificial Light at Night and Insomnia-Related Social Media Posts. *JAMA Netw Open*. Published November 20, 2024. doi:10.1001/jamanetworkopen.2024.46156

Data
Data available: Yes
Data types: Data (not involving human participants), Data dictionary
How to access data: The data that supports the findings of this study are available from the corresponding author upon reasonable request.
When available: With publication

Supporting Documents
Document types: Statistical/analytic code
How to access documents: The data that supports the findings of this study are available from the corresponding author upon reasonable request.
When available: With publication

Additional Information
Who can access the data: researchers whose proposed use of the data has been approved
Types of analyses: researchers whose proposed use of the data has been approved
Mechanisms of data availability: researchers whose proposed use of the data has been approved
